# PSMB7 Is a Key Gene Involved in the Development of Multiple Myeloma and Resistance to Bortezomib

**DOI:** 10.3389/fonc.2021.684232

**Published:** 2021-07-23

**Authors:** Dong Wu, Jiyu Miao, Jinsong Hu, Fangmei Li, Dandan Gao, Hongli Chen, Yuandong Feng, Ying Shen, Aili He

**Affiliations:** ^1^ Department of Hematology, The Second Affiliated Hospital of Xi’an Jiaotong University, Xi’an, China; ^2^ Department of Cell Biology and Genetics, Xi’an Jiaotong University Health Science Center, Xi’an, China

**Keywords:** multiple myeloma, proteasome inhibitor, PSMB7, WGCNA, drug resistance

## Abstract

Multiple myeloma (MM), the second most commonly diagnosed hematologic neoplasm, is the most significant clinical manifestation in a series of plasma cell (PC) dyscrasia. Monoclonal gammopathy of undetermined significance (MGUS) and smoldering MM (SMM), approximately 1% or 10% of which, respectively, can progress to MM per year, are the premalignant stages of MM. The overall survival (OS) of MM is significantly improved by the introduction of proteasome inhibitors (PIs), but almost all MM patients eventually relapse and resist anti-MM drugs. Therefore, it is crucial to explore the progression of MM and the mechanisms related to MM drug resistance. In this study, we used weighted gene co-expression network analysis (WGCNA) to analyze the gene expression of the dynamic process from normal plasma cells (NPC) to malignant profiling PC, and found that the abnormal gene expression was mainly concentrated in the proteasome. We also found that the expression of one of the proteasomal subunits PSMB7 was capable of distinguishing the different stages of PC dyscrasia and was the highest in ISS III. In the bortezomib (BTZ) treated NDMM patients, higher PSMB7 expression was associated with shorter survival time, and the expression of PSMB7 in the BTZ treatment group was significantly higher than in the thalidomide (Thai) treatment group. In summary, we found that PSMB7 is the key gene associated with MM disease progression and drug resistance.

## Introduction

Multiple myeloma (MM), accounting for 10% of all hematological malignancies, the final stage of a continuum plasma cell (PC) dyscrasia, which is characterized by malignant profiling of monoclonal protein with increased bone marrow (BM) PC, osteolytic lesion, hypercalcemia, and anemia (1). Monoclonal gammopathy of undetermined significance (MGUS) is a premalignant stage of MM that is defined as the presence of monoclonal immunoglobulin (Ig) in blood or urine (M protein), less than 10% clonal PC in the BM, and the absence of myeloma-related end-organ damage. Approximately 1% of MGUS patients evolve to MM per year (2, 3). MGUS may progress to a more advanced stage known as smoldering multiple myeloma (SMM). SMM, the probability of progression was 10% per years, is a transitional stage between MGUS and NDMM that is defined as M protein level of more than 3g/dl and/or >= 10% plasma cell in the BM without myeloma-related end-organ damage (4).

MM treatment has undergone many changes since the first well-documented case (5). In 1958, the introduction of melphalan was accompanied by alkylating agents and chemotherapeutic drugs, but overall survival did not significantly increase (6). In the 1980s, high-dose chemotherapy and autologous stem-cell transplantation (ASCT) were introduced, and subsequent prospective, randomized trial shown that high-dose therapy combined with ASTC could improve the response rate and overall survival in MM patients (7, 8). The introduction of immunomodulatory drugs (IMiDs) and proteasome inhibitors (PIs) expanded the therapeutic armamentarium for MM. Survival outcomes have significantly improved with the introduction of PIs, bortezomib, carfilzomib, and ixazomib, for the treatment of MM over the past decades (9). Despite an initially promising response, almost all MM patients eventually relapse and become refractory to anti-MM therapies due to drug resistance, which is the main obstacle in the treatment of MM. PIs drug resistance involves many mechanisms including increased endoplasmic reticulum (ER) stress, apoptosis failure, autophagy activation, aberrant expression of proteasomal subunits, and the bone marrow microenvironment (10–14). High-throughput genomic techniques have proved the existence of genomic instability and clonal heterogeneity during the pathogenesis of MM from the premalignant stage to the malignant proliferative stage (15). Thus, it is critical to determine the key biomolecules associated with drug resistance and related to the pathogenesis of normal PC to malignant proliferation PC.

In this study, based on WGCNA analysis of integrated dynamic progression datasets from health control (HC) to NDMM, we found that the expression of proteasome 20S subunit beta 7 (PSMB7), which could discriminate different stages of PC dyscrasia and positively correlated with the degree of the malignancy of PC dyscrasia. Additionally, the OS of NDMM patients with high expression of PSMB7 was shorter than that of patients with low expression of PSMB7 in the TT3 (total therapy 3) group and there was no statistical difference in the TT2 (total therapy 2) group and there was higher expression of PSMB7 in the TT3 than in the TT2 group.

## Materials and Methods

### Microarray Dataset

The microarray dataset were downloaded from the gene expression omnibus (GEO) database (https://www.ncbi.nlm.nih.gov/geo/). The MM international staging system (ISS) prognostication analysis dataset E-MTAB-4032 was downloaded from the ArrayExpress database (https://www.ebi.ac.uk/arrayexpress/experiments/E-MTAB-4032/) (16, 17). GSE6477 (https://www.ncbi.nlm.nih.gov/geo/query/acc.cgi?acc=GSE6477) was used to construct the co-expression network and evaluate the diagnostic value in different PC dyscrasia stages (18, 19). GSE24080 (https://www.ncbi.nlm.nih.gov/geo/query/acc.cgi?acc=GSE24080) was used to validate the a prognostic signature (20).

### Construction of Co-Expression Network

To construct a co-expression network by “WGCNA”, the original probeids in the GSE6477 dataset were converted to genesymbol (gene name). The gene expression level was defined as the average expression level when multiple probeids correspond to a specific genesymbol. The disease progression from HC to MGUS, SMM, and finally to NDMM was treated as a continuum. The soft threshold with the argument type “signed” is selected according to the scale-free (SF) topology criterion (21). A topological overlap matrix (TOM) was used to determine modules defined as clusters of densely interconnected genes (22). In order to quantify all of the genes on the array to every module, gene significance (GS) was defined as the correlation between the individual genes and the trait, and module membership was defined as the correlation between the module eigengene and the gene expression profile (23). Next, the modules and GS was related to the different stages of PC dyscrasias.

### GO and KEGG Pathway Enrichment Analysis

To identify the significant biological roles of the genes in the modules closely related to NDMM, gene ontology (GO) enrichment analysis and meaningful enrichment contained biological process (BP), molecular function (MF), and cellular component (CC) was performed using DAVID bioinformatics resources 6.8 (24). Pathway enrichment analysis was performed using the Kyoto Encyclopedia of Genes and Genomes (KEGG; http://www.Genome.Jp/kegg). KEGG pathway was selected with a cut‐off of a false discovery rate (FDR) of<0.05.

### Expression of PSMB7 in Different PC Dyscrasia Stages and ISS Statuses

The GSE47552 dataset, which contained 21 MGUS, 24 SMM, and 69 NDMM newly diagnosed without treatment patient samples and 15 NPC as health control (HC), was used to analyze the expression of PSMB7 in different PC dyscrasia stages. E-MTAB-4032, including 38 ISS I, 45 ISS II, and 69 ISS III, were used to analyze the expression of PSMB7 in different ISS statuses.

### c-BioPortal Analysis

Mutation analysis used MM datasets (Broad, Cancer Cell 2014), containing 203 multiple myeloma paired tumor/normal sample pairs, by the cBio Cancer Genomics Portal (http://cbioportal.org) (25–27).

### ROC Analysis

To measure the accuracy and specificity of PSMB7, the GSE6477 dataset was used for receiver operating characteristic (ROC) and area under the curve (AUC) analysis.

### Overall Survival Analysis

345 NDMM patients were treated with TT2 (TT2 added Thai to melphalan (MEL200)-based tandem autotransplants) and 214 NDMM patients were treated with TT3 (TT3 incorporated BTZ into a melphalan-based tandem transplant regimen) in GSE24080 was used for the OS analysis (28, 29). According to the average expression of PSMB7, the clinical and OS information of TT2 and TT3 treated NDMM patients was divided into the high or low expression group and the OS analysis was performed to calculate the correlation between the expression of PSMB7 and the survival time in TT2 or TT3.

## Results

### Construction of WGCNA and Identification of Modules

**Figure 1:**
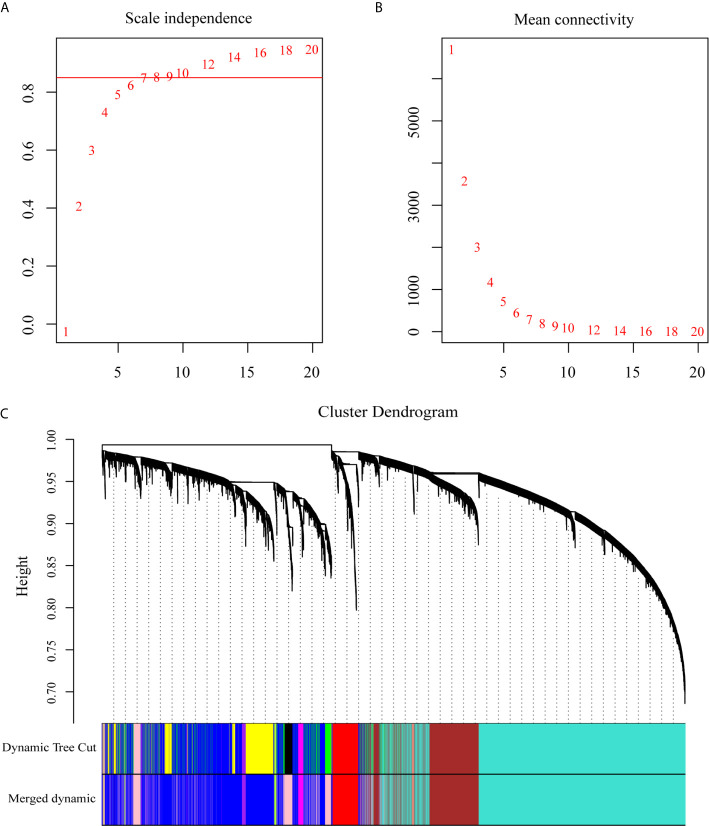
WGCNA on the dynamic progression of MM. **(A)** Analysis of network topology for various soft threshold powers (β). **(B)** Mean connectivity with various soft threshold power. **(C)** Clustering dendrogram of all differentially expressed genes with assigned module colors. Each color block represented a module of highly co-expressed genes.

To explore the dynamic changes associated with the PC dyscrasia process, we performed WGCNA analysis using the GSE6477 dataset, including HC, MGUS, SMM, and NDMM, showing the stepwise progression from the pre-MM stages to NDMM. The raw files were pre-processed by means of background correction and normalization, and the average gene expression was treated as the final gene expression (30, 31). A total of 13,236 genes was finally used for the WGCNA analysis. Soft threshold power β=8 was chosen for the adjacency matrix calculation and the SF topology fit index reached 0.85 (**Figures 1A, B**). Then, 12 gene modules were identified through TOM-based clustering (**Figure 1C**).

### Correlation Between the Module and Different PC Dyscrasia Stages

**Figure 2:**
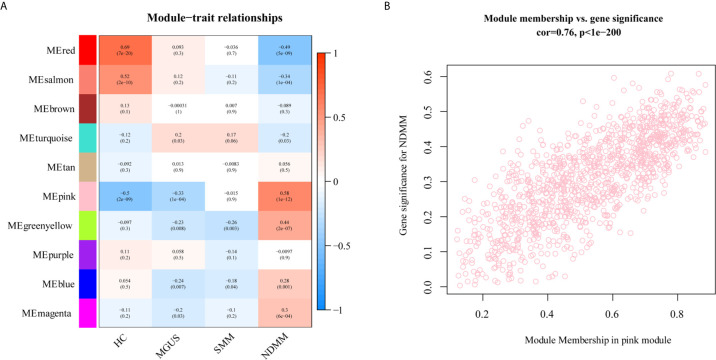
The relationship between modules and PC dyscrasia. **(A)** Module-trait associations. Each row corresponds to a ME, each column to a stage of PC dyscrasias. The table is color-coded by the corresponding correlation according to the color legend. **(B)** A scatterplot of GS for weight *vs.* module membership in the ME pink.

To explore the association between each module and different PC dyscrasia stages, eigengenes, the first principal component of the expression matrix of the corresponding module, was correlated to them (23). We noticed that the module eigengene (ME) pink was closely related to NDMM (**Figure 2A**). The GS and module membership were highly correlated (cor=0.076), illustrating that genes significantly associated with NDMM were also critical elements of ME pink (**Figure 2B**).

### Functional Enrichment Analysis Found That Proteasome Played a Crucial Role in the Development of MM

**Figure 3:**
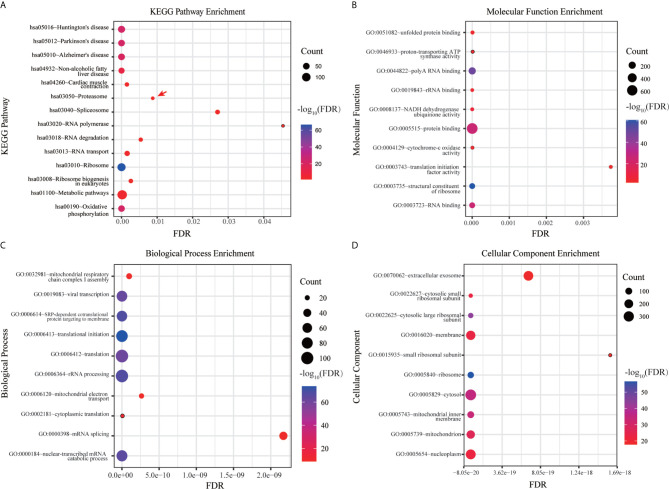
Functional enrichment analysis of hub genes. **(A)** KEGG pathway enrichment analysis. **(B)** MF enrichment analysis. **(C)** BP enrichment analysis. **(D)** CC enrichment analysis.

DAVID database were used to analyze the canonical pathway analysis and biological function of the genes contained in ME pink. As shown in **Figure 3A**, hsa03050: proteasome (red arrow) was mainly changed, suggesting that the proteasome changed significantly as the disease progressed. MF, BP, and CC enrichment analysis in ME pink was shown in **Figures 3B–D**.

### PSMB7 Discriminated Different PC Dyscrasia Stages and Its Expression Was the Highest in the ISS III Status of NDMM

**Figure 4:**
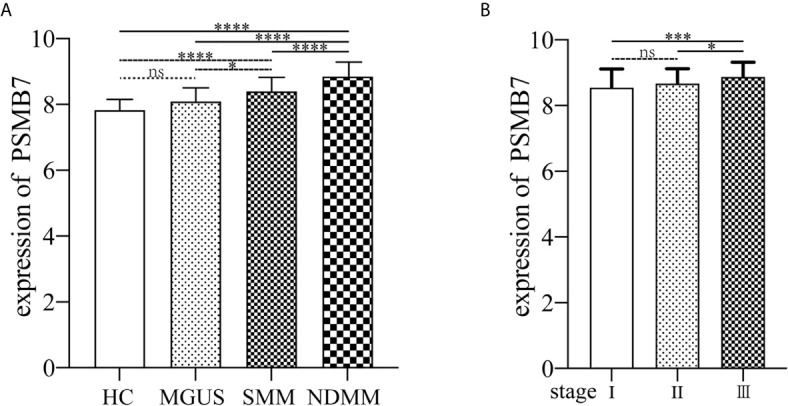
The expression of PSMB7. **(A)** The expression of PSMB7 from HC to NDMM in GSE6477. **(B)** The expression of PSMB7 in different ISS status in E-MTAB-4032. ns, not significant, *P < 0.05, ***P < 0.001, and ****P < 0.0001.

The GSE6477 dataset was used for explore the expression of genes in the proteasome at different PC dyscrasia stages. The results showed that PSMB7 could distinguish all stages of PC dyscrasia, from MGUS to SMM and NDMM (**Figure 4A**), HC *vs.* MGUS, *P*=0.112, HC *vs.* SMM, *P*<0.0001, HC *vs.* NDMM, *P*<0.0001; MGUS *vs.* SMM, *P*=0.019, MGUS *vs.* NDMM, *P*<0.0001; SMM *vs.* NDMM, *P*<0.0001. The E-MTAB-4032 dataset was used to explore the expression of PSMB7 in different ISS statuses of NDMM. The results showed that the expression of PSMB7 at ISS III was significantly higher than at ISS stage I (*P* = 0.001) and II (*P* = 0.038) in NDMM patients (**Figure 4B**).

### No Mutation Was Found in PSMB7

**Figure 5:**
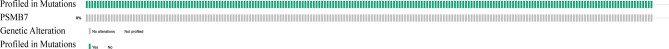
Mutation analysis of PSMB7 in NDMM. There were 0% genomic alterations of PSMB7 in MM.

Since the expression of PSMB7 is highest expressed in NDMM and ISS III, we wondered about the relationship between the high expression of PSMB7 and genomic alterations. Using cBioPortal to determine the types and frequency of PSMB7 alterations in 203 MM patients, no mutations were found, suggesting that the expression level of PSMB7 played a crucial role (**Figure 5**).

### PSMB7 Has the Highest Sensitivity and Specificity for NDMM

**Figure 6:**
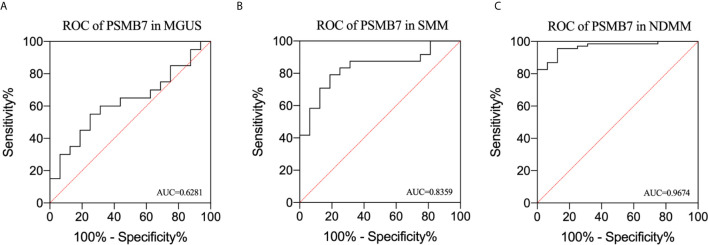
Receiver operating characteristic curve analysis in MGUS, SMM, and NDMM. **(A)** ROC of PSMB7 in MGUS, **(B)** ROC of PSMB7 in SMM, **(C)** ROC of PSMB7 in NDMM.

We performed ROC analysis of MGUS, SMM, and NDMM to analyze the risk signature of PSMB7. The AUC of MGUS, SMM, and NDMM are 0.628 (*P*=0.1918, 95%CI: 0.4445 -0.8118), 0.9028 (*P*=0. 95%, 95%CI: 0.7092-0.9626), and 0.9674 (*P*= <0.0001, 95% CI: 0.7092-0.9626),respectively, demonstrating that PSMB7 could discriminate HC from PC and NDMM had the highest sensitivity and specificity (**Figures 6A–C**).

### High Expression of PSMB7 With Shorter Overall Survival in TT3

**Figure 7:**
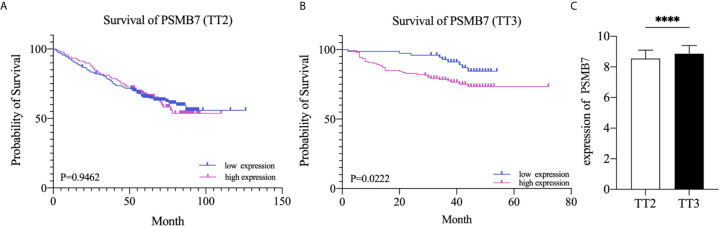
Kaplan-Meier survival curves of NDMM patients. **(A)** Overall survival of PSMB7 in the TT2 group. **(B)** Overall survival of PSMB7 in the TT3 group. **(C)** Expression of PSMB7 in the TT2 and the TT3 group, ****P < 0.001.

To further explore the relationship between the expression of PSMB7 and the clinical outcome in NDMM patients, the GSE24080 dataset, containing two kinds of MM chemotherapy: TT2 and TT3, was selected for OS analysis by means of Kaplan-Meier Survival analysis. The main difference between TT2 and TT3 was that TT2 contained Thai, and TT3 contained BTZ. The result suggested that the OS of PSMB7 in the TT3 low-expression group was significantly longer than that of TT3 high-expression group, and there was no difference in the TT2 group (**Figures 7A, B**). In addition, the expression of PSMB7 in the TT3 was significantly increased compared to the TT2 group (*P* < 0.001) (**Figure 7C**).

## Discussion

The malignant transformation from healthy cells to cancer cells is a multi-step and multi-faceted process. Cancer biology have shown that clonal evolution drives tumor development (15). Previous studies have shown that the proportion of PC in the BM and the rate of increase of the serum M-protein level during the first years can be used to predict the possibility of MGUS/SMM progressing to NDMM[ (32). Most of these studies focused on the differential gene expression in two stages of PC malignant transformation, such as comparing HC, MGUS, and SMM with NDMM, but the malignant transformation from HC to NDMM is a dynamic progression and these findings could not explain the underlying causes of the malignant progression.

In this study, by WGCNA, a systems biology algorithm can be applied to describing the correlation patterns among genes across microarray samples, finding highly related gene modules and relating modules to a particular PC dyscrasia stage (23). We analyzed the entire dynamic disease process from HC to NDMM and found that the abnormal gene expression of PC concentrated in the 26S proteasome, of which PSMB7 is positively correlated with the degree of PC malignancy and is highest expressed in NDMM. The proteasome is a large multi-catalytic protein complex that can degrade different kinds of cellular proteins (33). The 26S proteasomes is composed of 20S core particles (CP) and 19S regulatory particles (RP). 20S CP is a hollow structure in the middle, while 19S RP is located at both ends. 19S RP can recognize and bind ubiquitinated proteins and transport them to the core particle to degrade excess and abnormally folded proteins (34, 35). The proteasome and ubiquitin proteasome system (UPS) participate in the regulation of cell growth and survival. In the course of MM, abnormal UPS function can lead to excessive proteasome activation, which leads to excessive degradation of tumor suppressor p53 and the inhibition of nuclear factor-κB (NF-κB). The excessive degradation of various cancer suppressor factors and the activation of cancer-related pathways lead to the activation of NF-κB downstream effectors and form a positive feedback loop, enhancing the cell signal transmission in the BM microenvironment and the survival rate of MM cells, ultimately leading to the quick malignancy profiling of myeloma cells (36, 37).

PIs are an important new class of drugs for the treatment of multiple myeloma. The success of PIs is due to the susceptibility of myeloma cells to the inhabitation of the 26S proteasome (38). The 20S proteasome contains seven different subunits, the most important of which are PSMB6 (β1), PSMB7 (β2), and PSMB5 (β5). When stimulated by cytokine signals, the cellular stress response promotesβ1, β2, and β5 to convert into β1i, β2i, and β5i (39). The first generation of PIs, agent BTZ, is a dipeptide borate that reversibly binds to β5, β1, and β5i subunits. It prevents the formation of transcribed blood vessels and the adhesion of PC by inhibiting NF-κB transcription factor IκB kinase and bone marrow stromal cells from promoting MM cell apoptosis (40). Although PI agents improve the survival and prognosis of MM patients, drug resistance has become the biggest obstacle to their clinical application. In a previous study, Ruud Oerlemans et al. found that not only the PSMB5 but also PSMB6 and PSMB7 subunits increased in BTZ resistant cell lines CEM-C7 and 8226 (41). Thus, we further analyzed all of the genes contained in the proteasome, and found that the expression of PSMB7 was positively correlated with the degree of PC dyscrasia, and could distinguish the different PC dyscrasia stages.

Given the importance of the proteasome in MM biology and therapy, we further explored the relationship between PSMB7 and ISS status of NDMM and found that the expression of PSMB7 in ISS III was significantly higher than in ISS I/II, which means that PSMB7 was not only positively correlated with the malignant degree of PC dyscrasia, and it was also correlated with the ISS status of NDMM. More importantly, in the BTZ treatment group, the OS of the PSMB7 high expression group was significantly shorter than that of the low expression group with the BTZ treatment, but there was no difference in the Thai treatment group. Moreover, the expression of PSMB7 was higher in the BTZ group than in the Thai treatment group, which means increasing PSMB7 accompanies BTZ therapy. Subsequently, the ROC analysis of PSMB7 in MGUS, SMM, and NDMM was conducted. These results suggested that PSMB7 could act as a reliable diagnostic and prognostic indicator in NDMM.

PSMB7, together with PSMB5 and PSMB6 composes the 20S proteolytic of the proteasome complex 26S. It has been believed that PSMB5 played a vital role in cell viability, but other publications demonstrated that protein degradation could be inhibited only by simultaneously inhibiting PSMB5 and PSMB6 or PSMB7. Chang-Xin Shi *et al.* found that PSMB7 depletion was lethal to MM cell lines, and expression levels of PSMB5, PSMB6, and PSMB7 increased in patients after BTZ treatment, which was consistent with our findings (42).

Collectively, in this study, based on integrated bioinformatic analysis, we demonstrated that PSMB7 is a key gene in MM development and resistance to BTZ therapy. Our findings may provide clues for elucidating the mechanisms of malignant transformation in PC dyscrasia and may help identify patients at high risk of progression and point to more precise therapeutic targets.

## Data Availability Statement

Publicly available datasets were analyzed in this study. This data can be found here: https://www.ebi.ac.uk/arrayexpress/experiments/E-MTAB-4032/ https://www.ncbi.nlm.nih.gov/geo/query/acc.cgi?acc=GSE6477 https://www.ncbi.nlm.nih.gov/geo/query/acc.cgi?acc=GSE24080.

## Author Contributions

DW and AH conceived and designed the study. DW, JM, FL, and YF collected and processed data. DG, HC, and YS prepared tables and figures. DW and JM drafted the manuscript. AH and JH revised the manuscript. All authors contributed to the article and approved the submitted version.

## Funding

This work was supported by National Science and Technology Major Project (2019ZX09301139).

## Conflict of Interest

The authors declare that the research was conducted in the absence of any commercial or financial relationships that could be construed as a potential conflict of interest.

## Publisher’s Note

All claims expressed in this article are solely those of the authors and do not necessarily represent those of their affiliated organizations, or those of the publisher, the editors and the reviewers. Any product that may be evaluated in this article, or claim that may be made by its manufacturer, is not guaranteed or endorsed by the publisher.
